# Efficacy and safety of diclazuril nanoemulsion in control of *Eimeria tenella* in broilers

**DOI:** 10.1186/s12917-024-04325-z

**Published:** 2024-10-29

**Authors:** Azza A. El-Sawah, Shawky M. Aboelhadid, El-Shymaa N. El-Nahass, Hassan E. Helal

**Affiliations:** 1 Department of Poultry and Rabbit Diseases, Faculty of Veterinary Medicine, Beni-Suef University, Beni-Suef, Egypt; 2 Department of Parasitology, Faculty of Veterinary Medicine, Beni-Suef University, Beni-Suef, Egypt; 3 Department of Pathology, Faculty of Veterinary Medicine, Beni-Suef University, Beni-Suef, Egypt; 4 Veterinary Medicine Department, Elhelal Veterinary Clinic, Fayoum, Egypt

**Keywords:** Chicks, Diclazuril, Nanoemulsion, *Eimeria tenella*, Growth performance, Biochemical parameters

## Abstract

**Background:**

Nanotechnology has the potential to reduce drug dosage while increasing efficacy; thus, the current work intends to synthesize diclazuril nanoemulsion and assess its performance against experimental coccidiosis in broilers.

**Methods:**

Diclazuril nanoemulsion (DZN) was formulated and characterized by zeta seizer and zeta potential. The formulated DZN was evaluated in vivo against *Eimeria tenella* infected chicks. DZN and DZ were used in 2 programs; therapeutic and prophylactic. A total of 210 one-day-old broiler chicks were distributed equally into six groups. The controls were negative uninfected untreated and positive infected untreated (G1 & G2). Therapeutic groups (G3 & G4) treated by DZ and DZN after appearance of the clinical signs of coccidiosis and continued for 5 days. Prophylaxis groups (G5 & G6) received DZ and DZN at 3 days before challenge and continued for 5 days after infection. The treatments dosages were 10 mg/mL for DZ of commercial origin and 2.5 mg/mL for the prepared DZN. All groups (except negative control) orally infected then followed up for clinical signs of coccidiosis, mortality rate, oocysts count, performance, hematological and biochemical parameters in addition to histopathological lesions.

**Results:**

The therapeutic groups showed that both treated groups (DZ and DZN) revealed similar results including good body weight gain, a low lesion caecal score, a low daily and total oocyst shedding count, and a low mortality rate. Regarding the biochemical parameters, all parameters were affected during infection then restored after the 12th day post infection. However, in the prophylactic groups, showed mild clinical signs and the blood pictures and biochemical parameters were nearly like the control negative without infection.

**Conclusion:**

DZN at a quarter dose of standard DZ produced the same outcomes as DZ at 10 mg/mL. Furthermore, DZN does not impair the typical safety of diclazuril in treated chicks.

## Introduction

Coccidiosis is one of the most significant protozoan diseases that impact poultry. It is caused by several Eimeria species including; *E. tenella*,* E. acervulina*,* E. necatrix*,* E. brunetti*,* E. maxima*,* E. mitis*,* and E. praecox* [1]. Due to high mortalities and weight losses from coccidiosis, the poultry industry suffers economic losses [2, 3]. Coccidiosis has far-reaching implications, including a significant economic impact on the global chicken sector [4]. Economic losses in the poultry business owing to avian coccidiosis exceed an annual deficit of US$ 13–14 billion [5, 6]. Furthermore, Coccidiosis is one among the gastrointestinal parasites that pose a real threat to profitable, sustainable chicken small-scale farms and backyards in Africa [7]. *Eimeria tenella* infected chicken caecal lining epithelium, causing bloody diarrhea, reduced feed intake, weight losses, and fatalities [8].

One of the feed additive coccidiostats permitted by European Union Regulation 1831/2003 is diclazuril, which is thought to be an effective, less toxic, and broad-spectrum coccidiostatic [9]. The triazine phenylacetonitrile compound diclazuril is a highly effective and broad-spectrum anticoccidial used for the prevention and treatment of coccidiosis in commercial chickens. Although diclazuril acts on intracellular stages, many studies have linked it to the disruption of respiratory chain enzymes like dihydrofolate reductase enzyme (DHFR) [10]. Diclazuril caused merozoites to undergo apoptosis, which significantly decreased the number of Eimeria developmental stages [11]. A small supplementation of diclazuril (1 mg/kg feed or 1 mg/L in drinking water) is thought to be an effective dose to prevent and treat chicken coccidiosis caused by *E. tenella*,* E. acervulina*, and *E. maxima* [12]. Drug resistance emerged as a result of the repeated use of chemoprophylaxis and anticoccidial feed additives in treating coccidiosis [13]. So, one of our study goals is to using diclazuril in nano emulsion form as a method product developing. Nanotechnology covered the study of materials with diameters between 1 and 100 nanometers since 1974. A nanometer is one billionth of a meter (measured in units of 10 –9 m), or “Nano” in the metric system [14]. Due to its capacity to improve the administration of pharmaceuticals, nanotechnology has lately gained substantial importance across a variety of industries, including veterinary care [15]. The nano-emulsion enhanced the bioavailability of anticoccidals like toltrazuril on other similar substances. Toltrazuril mixed nano micelle exhibited a significantly higher bioavailability (215%) than regular toltrazuril solution. Many nanoparticles have been employed as delivery systems for nanomedicine. It improved drug delivery, decreased used doses, and increased efficacy [16]. The resistance to diclazuril was reported [13].

The main aim of the current study was synthesis of diclazuril nanoemulsion and evaluation its efficacy even in low dose against experimental coccidiosis in broilers.

## Materials and methods

### Ethics

The experiment was conducted in accordance with the ethical guidelines and procedures established by the Faculty of Veterinary Medicine, Beni-Suef University, Egypt, under license number (2017-BSUV-11) in October 2017.

### Parasite

*Eimeria tenella* isolate was provided from parasitology department, faculty of veterinary medicine, Beni Suif University. This isolate was firstly propagated in 5 chicks to obtain sufficient oocysts for conduct this study. The birds were euthanized eight days after inoculation, and their cecal contents were recovered. The oocysts were concentrated, sporulated in 2.5% potassium dichromate, and kept in a refrigerator (2–5 °C) until used in the experimental infection [17].

### Experimental birds

A number of 210 one-day-old broilers of the Cobb breed were raised in a dedicated section of the Department of Poultry Disease at the Faculty of Veterinary Medicine, Beni-Suef University, Egypt, under standard circumstances for in vivo experiment. The raising system consists of individual pens in a battery caged structure with slit flooring. The feeding rations were supplied in three sequential different energy and protein concentrations: 23% starter, 21% grower, and 19% finisher. The rations were commercial feeds free from any antibiotics or anticoccidials to ensure that any results obtained were unrelated to any other factors other than experimentally evaluated materials. Feeding and drinking were freely. The lighting system was a 24-hour light program. The pens were pre-heated six hours before chick’s arrival to be obtaining a temperature of 33 °C. This brooding temperature 33 °C was conserved at this level avoiding decreased to be 28 °C and relative humidity 30–50% until the end of first week. Then, the brooding temperature decreased to be 30 °C from 7th day to 14th day and relative humidity 40–60% that continued to day 21 old then reduced to 25 °C to the end of experiment [18].

### Diclazuril forms

Pharma Swede Company in Cairo, Egypt provided the commercial form of diclazuril, Diclosol 1% (Diclazuril 10 mg/ml) utilized in the study.

## Diclazuril nanoemulsion preparation and characterization

Diclazuril nanoemulsion 0.5% was made in a 100 mL glass baker by dissolving 0.5 g of diclazuril powder in 20 mL of monopropylene glycol on a hot plate using a magnetic stirrer at 70 ^o^ C. After 5 min, 20 mL of triethanolamine and 5 mL of benzyl alcohol were added, with stirring for 10 min, complete the volume to 100 mL by distilled water. The emulsion was then transformed into a nanoemulsion in a second stage by being sonicated in a sonicator for five minutes, until a diclazuril nano emulsion had developed. Characterization of the nanoemulsion of diclazuril was done utilizing a Malvern zeta seizer nano series instrument.

### Experimental design

**Fig. 1:**
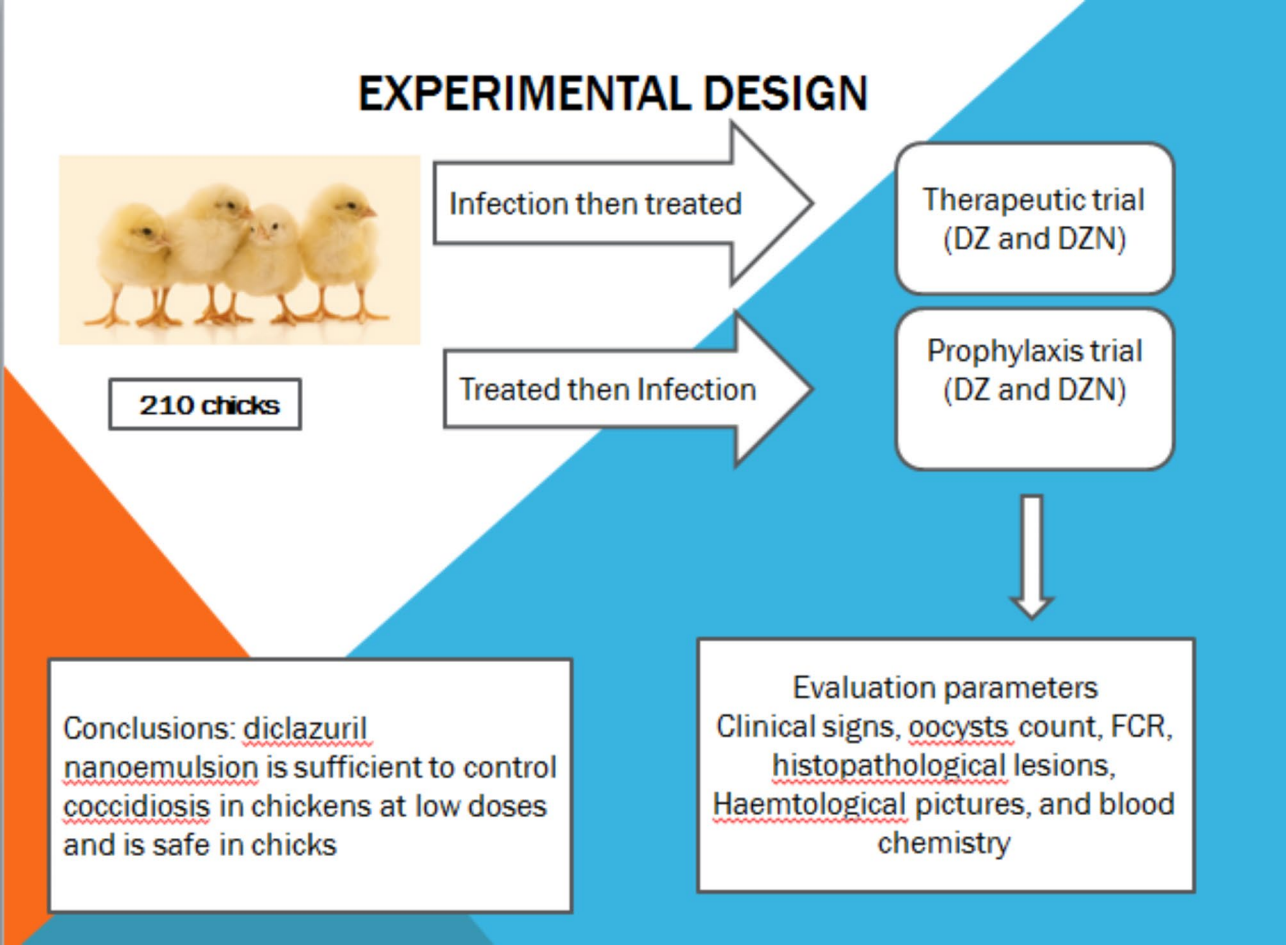
Experimental design of the work study

A total of 210 one-day-old chicks were used in the experiment, with 35 chicks (5 replicates of 7 in each) divided into six groups. Control groups were group 1 and group 2 (G1 & G2) negative uninfected untreated and positive infected untreated, respectively (Fig. 1). The treatments were applied into two programs; therapeutic and prophylaxis trials. The therapeutic trial composed of two groups; diclazuril commercial form (G3, 10 mg/liter) and diclazuril nanoemulsion (G4, 2.5 mg/liter). G3 and G4 were administered the treatments at five days post infection started with the clinical signs appearance at the day 5 post infection. The prophylaxis trial contained G5 that was administered diclazuril commercial form, 10 mg/liter, and G6 was administered diclazuril nanoemulsion, 2.5 mg/liter. The prophylaxis groups were administered the medications at three days before infection and continued five days post infection [18]. The chicks in all groups at the age of 23 days, with the exception of G1, were experimentally orally infected with 25 × 10^3^ sporulated oocysts of *E. tenella*, and were monitored daily for observation. For each group of chicks, clinical signs, body weight, feed consumption, and feed conversion rate were recorded. At days 6th, 9th, and 12th post infections, five birds (a bird from each replicate) from each group were randomly chosen and humanely sacrificed by manual cervical dislocation. The caeca were taken for histopathology, and the blood samples were taken for hematological and biochemistry analysis. From the sixth day after infection until the 10th day after infection, five random fecal samples were taken daily. These samples were used to calculate the daily rate of oocyst shedding.

## Evaluation parameters

### Clinical parameters

The clinical sings of caecal coccidiosis, mortality rate, bloody diarrhea score, and caecal lesion score were recorded [19, 20].

### Parasitological parameters (Oocyst shedding count)

Daily collection of five pooled feces samples from the replicates for each group, were made from each of the four corners and from the cage’s center [21]. The collected feces were thoroughly combined in a plastic sterilized cup and stored in the refrigerator. One gram of the combined fecal samples was then ten-diluted by saturated salt solution, counted on a McMaster slide, and the daily oocyst number was recorded in grams of faces three times for each group. This process was repeated from day 6 till the day 10 PI.

### Performance indicators

The growth rate was measured by weighing each group’s individual birds at the beginning of the experiment and repeating the weigh-in every week [22]. The formula for calculating the feed conversion ratio (FCR) was as follows: FCR = total weight (gram) of consumed feed of each group of birds during a specific period/total weight increase (g) of the same birds, even the dead birds throughout the same period [23]. Additionally, carcass cut was done for each group at the end of the experiment.

### Histopathology examination

Five caecal tissue samples were used for histopathology for each group. The collecting small pieces of caecal tissue were put in 10% formalin, washing them in tap water overnight, dehydrating then encasing them in paraffin wax and cutting them into paraffin sections with a thickness of 5 microns and then rehydrated five more times, each time using deionized water with ascending concentrations of alcohol. Haematoxylin and eosin (H & E) stain was used to stain the histological Sect. [24].

## Safety of the used DZN

### Hematological parameters

Hematological parameters were measured using two techniques; the first using a CBC automation veterinary analyzer and the second using blood film stained with eosin and methylene blue stains. Five samples from each group were collected at the sixth, ninth, and twelfth day following infection using each method.

### Blood chemistry parameters

Five blood samples were taken from each group, and the serum was obtained by centrifuging the samples at 3000 rpm for roughly 10 min. Blood chemistry measurements made by Fuji Dry Chem automatically. By taking three samples for each group at specific intervals of days 6, 9 and 12 following infections, a full automation system can assess the liver enzymes and renal enzymes. By measuring liver enzymes Aspartate Aminotransferase (AST) or serum glutamic oxaloacetic transaminase (GOT) and Alanine Aminotransferase (ALT) or serum glutamic pyruvic transaminase (GPT), as well as total protein (TP) [25].

### Statistical analysis

The Shapiro-Wilk test was used to determine whether the distribution of the data was normal. To ascertain differences between groups, data were later analyzed using an ANOVA and a Tukey multiple range test (Graph pad Software, San Diego, CA). The findings are presented as mean SD. Statistical significance was defined as a probability value of 001 (*P* ≤ 0.001).

## Results

### Diclazuril nanoemulsion (DZN) characterization

**Fig. 2:**
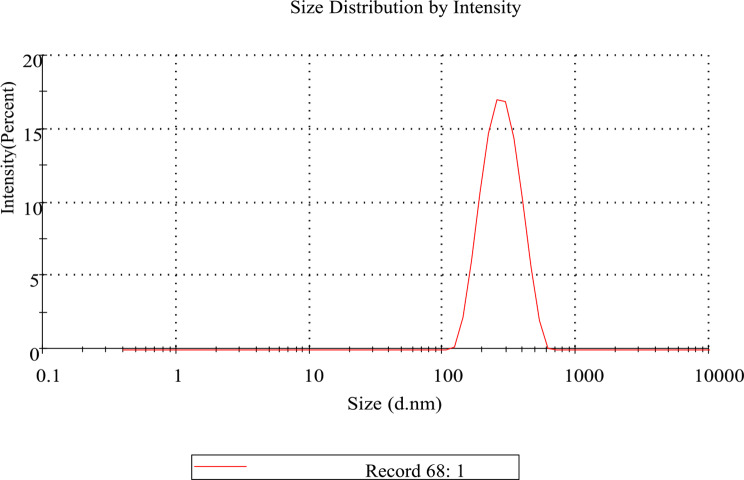
Diclazuril nanoemulsion droplet size

**Fig. 3:**
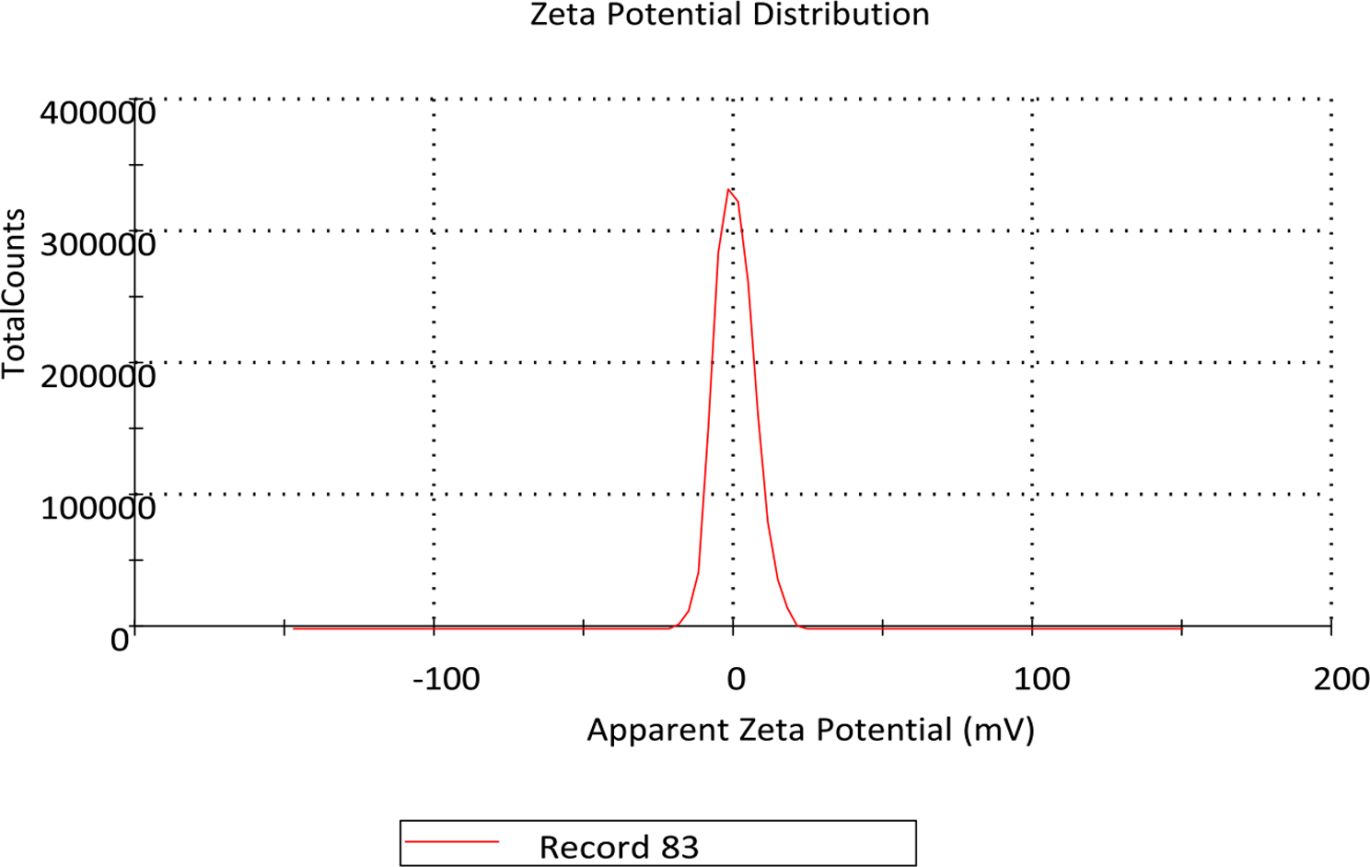
Diclazuril nanoemulsion zeta potential distribution

Zeta apparatus measurements revealed information about potential measurements that had positive charge on the outer surface of DZN (0.0981 mV) and the hydrodynamic particle size of 394 d.nm, with PDI = 0.325 d.nm. The low value of PDI (< 1.00) reflected the homogeneous size distribution of the droplets (Figs. 2 and 3).

### Evaluation parameters

#### Clinical signs

The uninfected untreated control group was free of infection and had no clinical indications of cecal coccidiosis or any other coccidiosis. At the fifth day following infection, bloody diarrhea, ruffled feathers, lower feeding rate, and vitality reduction were highly evident in infected untreated group (G2). These signs appeared in the therapeutic groups G3 and G4, but at less severity. The score for bloody diarrhea was noted in Table 1. Moreover, the uninfected untreated control group had the lowest mortality rate (3%). However, G2 had the highest mortality rate (33%). The therapeutic groups showed the same mortality of 31.42%. However, prophylaxis groups (G5 and G6) showed slight bloody diarrhea (score 1) associated with mild clinical signs and low mortality rate 3%.

**Table 1:** Bloody diarrhea score in the therapeutic and prophylaxis groups treated by diclazuril ordinary and nano forms at day 7 post infection

Day/Group	6th dpi	7th dpi	8th dpi	9th dpi
Group 1 (uninfected untreated)	0 (score 0)	0 (score 0)	0 (score 0)	0 (score 0)
Group2 (infected untreated)	++++ (score 4)	+++ (score 3)	+ (score 1)	0 (score 0)
Group 3 (Diclazuril 10 ppm) treatment	++++ (score 4)	++ (score 2)	+ (score 1)	0 (score 0)
Group 4 ( Nano Diclazuril 2.5 ppm) treatment	++++ (score 4)	+ + (score 2)	+ (score 1)	0 (score 0)
Group 5 (Diclazuril 10 ppm) prophylaxis	+ (score 1)	0 (score 0)	0 (score 0)	0 (score 0)
Group 6 ( Nano Diclazuril 2.5 ppm) prophylaxis	+ (score 1)	0 (score 0)	0 (score 0)	0 (score 0)

Bloody diarrhea score in treated and un treated groups: + means 0–25% blood in the feces, ++ means 50% blood in the feces, +++ means 75% blood in the feces and ++++ means 100% blood in the feces

G5 and G6 showed slight bloody diarrhea (score 1) associated with mild clinical signs

#### Parasitological count of oocysts shed in the feces

The use of DZ and DZN as prophylactic treatment revealed lower number of oocysts in compared to therapeutic groups. No significant difference between prophylactic groups was reported. However, the therapeutic groups showed lower number of oocyts count in a significant manner in compared with control infected untreated group (Table 2). At day 10 PI, the count of oocysts sharply declined in the treated groups to reach 30 × 10³ and 27 × 10³ in DZN and DZ respectively, while the count in G2 relatively still higher than treated groups, recording 105 × 10³ opg (Table 2).

**Table 2:** Oocyst count per gram feces at different days in the therapeutic and prophylaxis groups treated by diclazuril ordinary and nano forms

Day/ Group	Oocyst count (10^3^) (Mean ± SD)			
	Day 7	Day 8	Day 9	Day 10
Group 1 (uninfected untreated)	0.00 ± 0.00	0.00 ± 0.00	0.00 ± 0.00	0.00 ± 0.00
Group2 (infected untreated)	165 ± 47.7	575 ± 137	206 ± 56.2	105 ± 17.3
Group 3 (Diclazuril 10 ppm) treatment	72.0 ± 15.4*	105 ± 37.4*	41.0 ± 11.5*	27.0 ± 12.3*
Group 4 ( Nano Diclazuril 2.5 ppm) treatment	65.0 ± 22.6*	125 ± 49.5*	34.0 ± 10.1*	30.0 ± 13.1*
Group 5 (Diclazuril 10 ppm) prophylaxis	9.00 ± 1.00*	36.0 ± 8.54*	22.0 ± 3.00*	6.67 ± 1.53*
Group 6 ( Nano Diclazuril 2.5 ppm) prophylaxis	10.3 ± 3.06*	47.3 ± 15.7*	25.7 ± 5.86*	8.67 ± 0.58*

(*) significant for control positive (infected untreated), *P* ≤ 0.05, (DZN) Diclazuril nanoemulsion infected treated, (DZ) Diclazuril infected treated, Control negative (Non-infected non-treated)

#### Performance of treated chicks, feed conversion rate (FCR), growth rate and feed consumption

Before infection, all groups had similar FCR values ranging between 1.4 and 1.44. FCR was negatively impacted due to the infection and clinical signs. Eventually, at day 12 post infection, prophylaxis groups G5 and G6 recorded FCR similar to the control negative group (Table 3). However, the therapeutic groups; G3 and G4 reported an improvement in FCR. The untreated, uninfected group G1 had the best FCR, while, a moderate growth rate was achieved in infected treated groups. A poor growth rate was reported in the infected untreated group; however, G1, G5, and G6 recorded the highest growth rate (Table 3). Due to the challenge, the growth rate ceased definitively five days after infection and returned gradually up to ninth day post-infection, and semi-totally recovered at the end of the experiment at 12th day post infection (35-day-old broilers). The percent of the increase in body weight gain showed the difference between groups as the uninfected untreated group recorded 99.31%, prophylaxis group G5 98.03%, prophylaxis group G6 97.05%, therapeutic group G4 84.36%, therapeutic group G3 82.53%, while the infected untreated group recorded only 46.91% increase of B.W. gain. The carcass meat quality is very important in poultry industry that the most important end product is the part fed for human consumption and that table declared the importance of prophylaxis against coccidiosis that the control negative and prophylaxis groups G5 and G6 recorded the highest whole chicken weight (Table 4). The therapeutic groups G3 and G4 recorded the moderate in whole chicken weight. The infected untreated group recorded the worst whole chicken weight (Table 4).

**Table 3:** Feed consumption, body weight and feed conversion ratio in the therapeutic and prophylaxis groups treated by diclazuril ordinary and nano forms at day 12 post infection

Group	Body weight gain at the end of experiment (g)	% of increase of B.W. gain	FCR
G1 (uninfected untreated)	1009	99.31	1.56 ± 0.03
G2 (infected untreated)	479	46.91	2.1 ± 0.02
G3 (Diclazuril 10 ppm treatment)	841	82.53	1.65 ± 0.05
G4 ( Nano Diclazuril 2.5 ppm treatment)	858	84.36	1.7 ± 0.01
G5 (Diclazuril 10 ppm prophylaxis)	998	98.03	1.6 ± 0.03
G6( Nano Diclazuril 2.5 ppm prophylaxis)	990	97.05	1.62 ± 0.05

(*) significant for control negative (non- infected non-treated), *P* ≤ 0.05, (DZN) Diclazuril nanoemulsion infected treated, (DZ) Diclazuril infected treated, Control positive (infected non-treated)

**Table 4:** Chicken meat quality cuts at the end of the experiment in all groups

groups	Control negative	Control positive	DZ therapeutic	DZN therapeutic	DZ prophylaxis	DZN prophylaxis
Life body weight (Mean ± SD)	2026 ± 158	1510 ± 52.9	1868 ± 52.6*	1875 ± 173*	2016 ± 72.3*	2010 ± 165*
DE feathered carcass	1788 ± 129	1252 ± 42.0	1577 ± 25.1*	1504 ± 126*	1751 ± 61.7*	1745 ± 146*
Whole chicken without head and neck	1535 ± 148	977 ± 73.5	1298 ± 22.5*	1261 ± 130*	1451 ± 34.4*	1434 ± 119*
Breast Quarter	423 ± 60.1	263 ± 10.4	357 ± 24.7*	364 ± 40.9*	423 ± 9.29*	404 ± 29*
Breast Split	323 ± 46.4	198 ± 24.2	269 ± 4.04*	259 ± 33.4*	316 ± 21.5*	321 ± 22.9*
Leg Quarter	307 ± 16.1	201 ± 25.1	267 ± 22.5*	255 ± 26.7*	300 ± 10.4*	298 ± 24.0*
Liver	46.3 ± 0.58	42.0 ± 5.19	43.7 ± 2.88	43.3 ± 4.16	44.7 ± 1.15	43.3 ± 2.08
Heart	9.00 ± 1.73	8.42 ± 1.01	9.00 ± 0.00	9.00 ± 1.73	8.67 ± 0.58	8.23 ± 0.40
Spleen	2.89 ± 0.20	2.17 ± 0.29	2.01 ± 0.23	2.18 ± 0.28	2.13 ± 0.15	2.10 ± 0.17
Gizzard & proventriculus	40.7 ± 7.77	40.2 ± 1.76	43.8 ± 3.33	40.0 ± 1.00	43.8 ± 1.31	46.3 ± 1.62
Whole intestine	125 ± 5.77	147 ± 29.2	140 ± 16.8	122 ± 10.8	125 ± 1.15	126 ± 3.21
Caecum	16.3 ± 0.58	10.0 ± 2.64	12.5 ± 4.79	9.67 ± 1.53	11.3 ± 0.58	11.7 ± 2.08

(*) significant for control positive (infected untreated), *P* ≤ 0.05, (DZN) Diclazuril nanoemulsion infected treated, (DZ) Diclazuril infected treated, Control negative (Non-infected non-treated)

#### Post-mortem lesions and histopathological findings

**Fig. 4:**
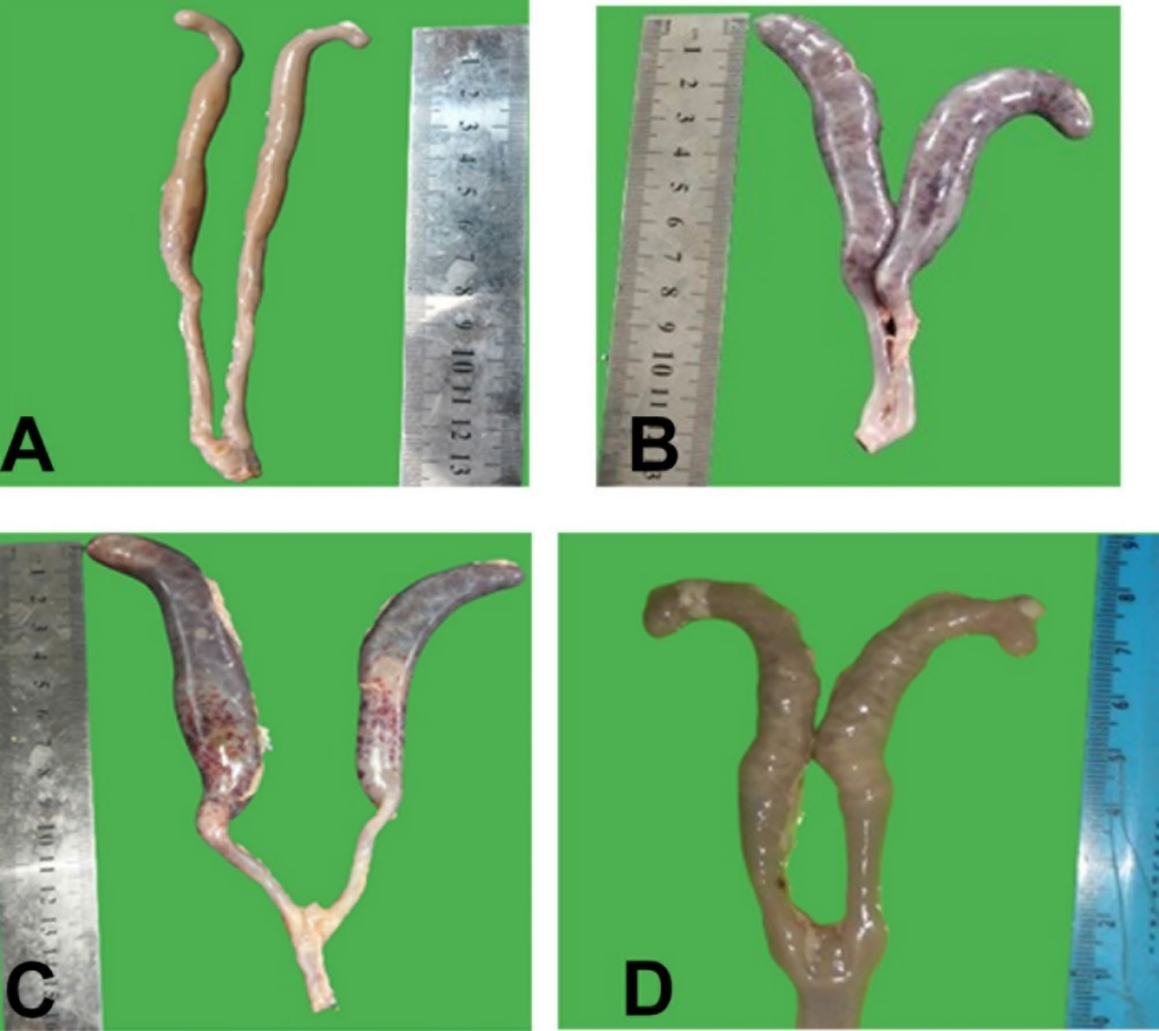
Post mortemum findings in the experimental groups. **A**. Negative control showed ceci without lesions. **B**. Positive control infected untreated group showed severe affected ceci, enlarged filled with blood. **C**. Ceci of therapeutic groups showed ceci with severe lesions in less degree than control positive. **D**. Ceci of prophylactic groups showed mild lesions

**Fig. 5:**
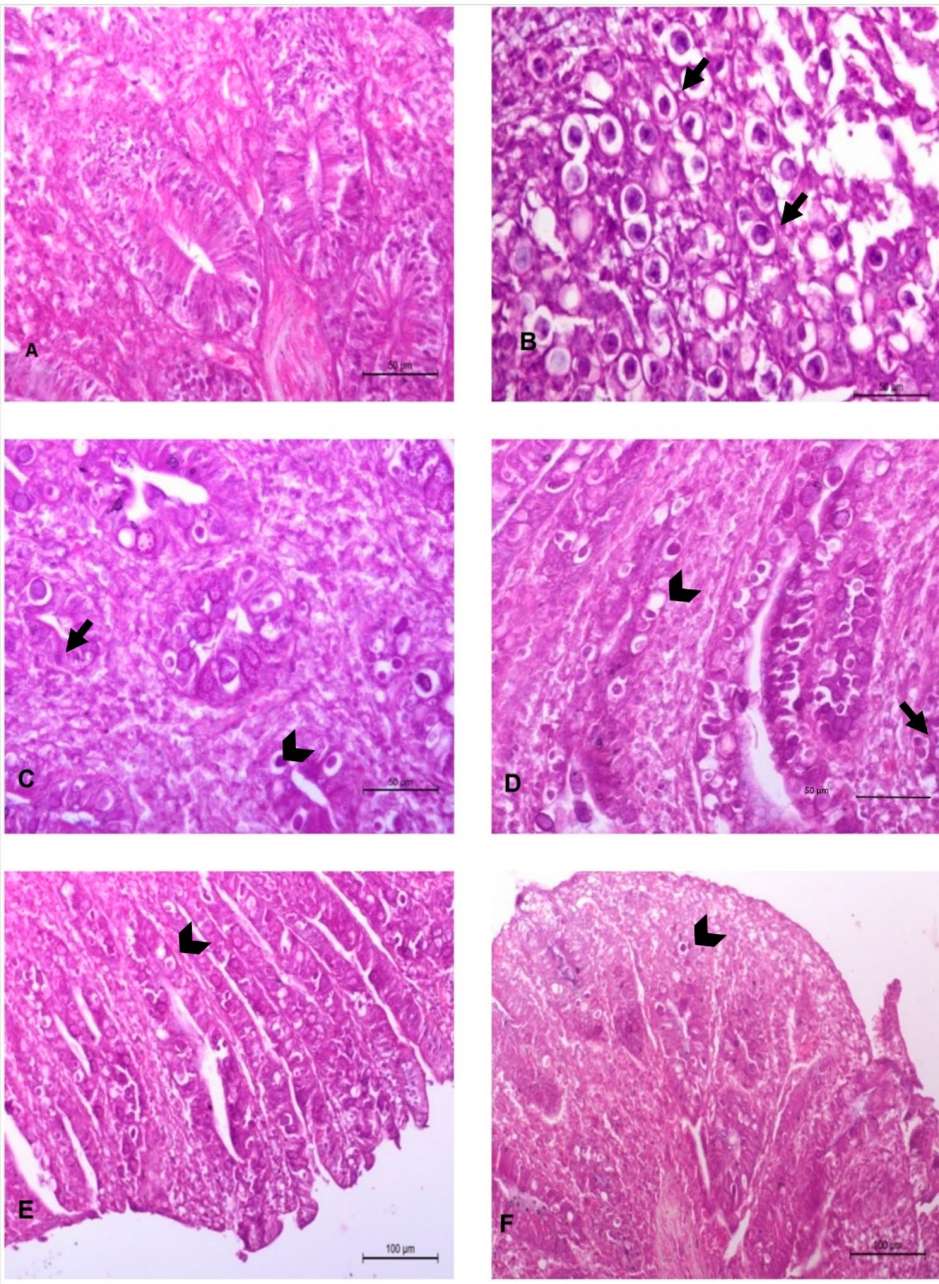
**A**. negative control appeared normal without any parasitic stages. **B**. Positive infected untreated group showed several numbers of parasitic stages (arrows). **C** & **D**. Therapeutic groups of diclazuril and DZN showed cecal tissue with mild infiltrated inflammatory cells (arrows), **D**. Therapeutic DZN group showed less number of parasitic stages (arrows heads). **E** & **F**. Prophylaxis groups; DZ (A) and DZN (B), low number of parasitic stages (arrow heads) and the epithelial lining some intact in compared to control infected untreated group

When applied a post mortem examination before the fifth day randomly showed no effect in any group. After the 5th day post infection, all infected groups showed affection except in G1 (all the organs were in a normal state, including the caecum). In G2 displayed typical cecal coccidiosis including severe caecal enlargement with a bloody caecal core. These lesions were observed in the therapeutic groups G3 and G4 but with less severity. In the prophylactic groups, the lesions were mild where the ceci contained mucus tinged with blood (Fig. 4). Moreover, the using of DZ or DZN are not adversely affected the liver texture or weight. Furthermore, post mortem examination in all groups showed normal duodenum, jejunum, ileum and rectum all over the experiment. However, G1 showed normal histologic intestinal villi, including caecal lining epithelium which showed normal caecal mucosa, submucosa, musculosa, and serosa. The chicks in G2 showed severe congestion and coagulative necrosis of lining epithelium. Sever villous atrophy with epithelial cells of microvilli intruded with undifferentiated and differentiated gamonts, macrogametocytes and microgametocytes with infiltration of eosinophils, mononuclear cells and fibriocytes. Oocysts invaded mucosa and extend to the submucosa and tunica muscularis and aggregated in form of clusters (Fig. 5B). However, the caecal walls of therapeutic chicks in G3 and G4 had significantly fewer eimerian developmental stages than in the control infected untreated group (Fig. 5C&D). Prophylaxis groups G5 and G6 revealed low number of parasitic stages and the epithelial lining some intact in compared to control infected untreated group (Fig. 5E &F).

## Safety of the used DZN

### Liver and kidney functions tests

It was observed that creatinine, urea and total protein were reserved to their normal levels of renal enzymes at the end of the experiment (12 days post infection). The liver functions showed that GPT was in normal level in all groups and significant difference between the groups. Otherwise, the infected groups had higher level of glutamic oxaloacetic transaminase (GOT) than control uninfected untreated group (Table 5).

**Table 5:** Blood chemistry at day 12 post infection in the therapeutic and prophylaxis groups treated by diclazuril ordinary and nano forms

Parameters	Control negative	Control positive	DZ therapeutic	DZN therapeutic	DZ prophylaxis	DZN prophylaxis
Creatinine	1.43 ± 0.22	1.91 ± 0.24	2.19 ± 0.52*	1.34 ± 0.14	1.50 ± 0.20	1.20 ± 0.60
Blood urea nitrogen	2.48 ± 0.18	2.89 ± 0.17	2.72 ± 0.14	2.54 ± 0.03	2.73 ± 0.25	2.70 ± 0.20
GPT	25.5 ± 0.46	27.0 ± 2.96	25.2 ± 1.45	25.4 ± 0.54	25.7 ± 1.53	24.8 ± 1.26
GOT	50.0 ± 30.4	179 ± 7.77	174 ± 26.1*	145 ± 19.0*	143 ± 7.64*	135 ± 4.51*
Total protein	1.80 ± 0.04	1.96 ± 0.06	1.81 ± 0.03	2.01 ± 0.08*	1.83 ± 0.05	1.91 ± 0.18

(*) significant for control negative (non- infected non-treated), *P* ≤ 0.05, (DZN) Diclazuril nanoemulsion infected treated, (DZ) Diclazuril infected treated, Control positive (infected non-treated)

### Hematological parameters

At day 6 post infection, CBC profile of groups; G2, G3 and G4 revealed decreased count of red blood cells, level of hemoglobin and hematocrit which indicate sever hemorrhage due to the infection. There were increased count of MID cells (eosinophil, basophil and monocytes) especially eosinophil count that means parasitic infection (Table 6). At day 8 post infection, RBCs, HGH and HCT started to increase and become nearly normal because stop bleeding and high platelets count due to coagulation and healing of infected cecal tissues (Table 7). At day 12 post infection, the hematological picture restored to its normal levels and the platelets count started to be nearly normal (Table 8). In case of DZN and DZ prophylaxis, at day 6 PI, CBC profile detected normal WBC cells with high lymphocyte cells, and slightly decrease RBCs count, HGB and HCT. At day 12 PI, nearly normal blood picture was determined (Table 8).

**Table 6:** Blood picture at day 6 post infection in the therapeutic and prophylaxis groups treated by diclazuril ordinary and nano forms

Parameters/Groups	Control negative	Control positive	DZ therapeutic	DZN therapeutic	DZ prophylaxis	DZN prophylaxis
WBCs	39.7 ± 2.52	59.7 ± 4.56	64.3 ± 5.86*	60.7 ± 10.4*	38.9 ± 4.60	42.6 ± 4.66
Granulocytes	56.5 ± 4.82	44.7 ± 0.64	46.0 ± 9.64*	43.5 ± 5.22*	33.7 ± 6.14*	33.4 ± 3.97*
Lymphocytes	31.1 ± 2.59	31.2 ± 1.07	33.3 ± 4.04	34.3 ± 4.16	52.2 ± 5.48*	52.1 ± 3.54*
MID	12.4 ± 2.25	24.0 ± 1.70	18.3 ± 7.51	22.0 ± 3.61*	14.1 ± 0.66	14.5 ± 0.70
RBCs	2.41 ± 0.04	0.80 ± 0.00	0.88 ± 0.04*	0.89 ± 0.01*	2.19 ± 0.15*	2.23 ± 0.07*
HGB	9.92 ± 0.13	3.78 ± 0.02	3.81 ± 0.08*	3.78 ± 0.08*	8.83 ± 038*	9.23 ± 0.21*
HCT	25.8 ± 0.25	9.24 ± 0.21	9.54 ± 0.49*	9.29 ± 0.23*	21.6 ± 0.85*	22.4 ± 0.69*
Platelets	2.83 ± 0.29	3.25 ± 0.25	3.70 ± 0.85	3.20 ± 0.44	5.00 ± 2.00*	1.67 ± 0.58

(*) significant for control negative (non- infected non-treated), *P* ≤ 0.05, (DZN) Diclazuril nanoemulsion infected treated, (DZ) Diclazuril infected treated, Control positive (infected non-treated), (MID) indicates the combined value of the other types of white blood cells not classified as granulocytes or lymphocytes (such as eosinophils and basophils and monocytes)

**Table 7:** Blood picture at day 9 post infection in the therapeutic and prophylaxis groups treated by diclazuril ordinary and nano forms

Parameters/Groups	Control negative	Control positive	DZ therapeutic	DZN therapeutic	DZ prophylaxis	DZN prophylaxis
WBCs	84.4 ± 12.8	105 ± 17.2	80.8 ± 2.25	73.3 ± 0.25	46.3 ± 4.57*	52.7 ± 3.71*
Granulocytes	60.2 ± 7.48	66.6 ± 2.31	59.5 ± 2.50	58.3 ± 1.89	36.9 ± 0.85*	38.0 ± 2.98*
Lymphocytes	29.0 ± 5.89	19.2 ± 6.41	29.9 ± 1.85	31.2 ± 1.36	49.1 ± 0.34*	47.4 ± 2.73*
MID	10.8 ± 1.59	14.2 ± 4.09	10.6 ± 0.66	10.5 ± 0.53	14.0 ± 0.80	14.6 ± 0.25*
RBCs	2.58 ± 0.14	1.55 ± 0.21	1.73 ± 0.08	1.97 ± 0.01*	2.25 ± 0.26*	2.46 ± 0.04
HGB	11.1 ± 0.68	7.43 ± 0.21	7.43 ± 0.15*	7.90 ± 0.17*	9.13 ± 0.61*	10.3 ± 0.15*
HCT	29.4 ± 1.35	19.6 ± 1.50	20.9 ± 0.76*	22.1 ± 0.29*	21.7 ± 1.56*	24.6 ± 0.46*
Platelets	3.23 ± 0.64	44.7 ± 1.15	47.7 ± 2.31*	41.7 ± 2.08*	4.00 ± 4.36	13.0 ± 2.00*

(*) significant for control negative (non- infected non-treated), *P* ≤ 0.05, (DZN) Diclazuril nanoemulsion infected treated, (DZ) Diclazuril infected treated, Control positive (infected non-treated), (MID) indicates the combined value of the other types of white blood cells not classified as granulocytes or lymphocytes (such as eosinophils and basophils and monocytes)

**Table 8:** Blood picture at day 12 post infection in the therapeutic and prophylaxis groups treated by diclazuril ordinary and nano forms

Groups / parameters	Control negative	Control positive	DZ therapeutic	DZN therapeutic	DZ prophylaxis	DZN prophylaxis
WBCs	68.7 ± 6.80	70.9 ± 5.66	80.2 ± 7.48*	75.0 ± 4.74	50.8 ± 5.27*	49.9 ± 5.23*
Granulocytes	56.9 ± 6.59	56.1 ± 2.08	60.1 ± 6.46	59.9 ± 1.86	40.0 ± 4.68*	36.8 ± 3.39*
Lymphocytes	31.4 ± 5.90	32.3 ± 1.78	29.2 ± 4.91	29.6 ± 1.63	46.1 ± 3.96*	48.7 ± 3.13*
MID	11.7 ± 0.70	11.6 ± 0.31	10.7 ± 1.55	10.5 ± 0.26	13.9 ± 0.76*	14.5 ± 0.26*
RBCs	2.56 ± 0.21	2.53 ± 0.11	2.51 ± 0.09	2.38 ± 0.59	2.12 ± 0.21	2.36 ± 0.26
HGB	10.6 ± 1.21	9.63 ± 0.57	10.0 ± 0.10	9.70 ± 1.22	8.83 ± 0.71	9.37 ± 0.76
HCT	28.8 ± 2.93	27.7 ± 2.51	29.4 ± 1.46	25.8 ± 3.53	20.9 ± 1.97*	22.1 ± 1.87*
Platelets	2.57 ± 0.12	15.3 ± 2.31	18.0 ± 3.61*	19.3 ± 6.35*	3.67 ± 2.88	2.00 ± 0.00

(*) significant for control negative (non- infected non-treated), *P* ≤ 0.05, (DZN) Diclazuril nanoemulsion infected treated, (DZ) Diclazuril infected treated, Control positive (infected non-treated), (MID) indicates the combined value of the other types of white blood cells not classified as granulocytes or lymphocytes (such as eosinophil and basophils and monocytes)

## Discussion

In the therapeutic trial, the chicks in groups G3 and G4 firstly showed clinical and recovered quickly. The clinical signs that caused by emerian sporozoites liberation and penetration in the intestinal mucosal cells [26]. The success of DZ forms in coccidiosis control is not a surprise where DZ showed efficacy against different species of Eimeria species [27–30]. Earlier, Since DZ was a new anticoccidial in 1989, the lowest doses were documented with good results for the prevention of major *Eimeria* species infections, including *E. acervulina*,* E. necatrix*,* E. maxima*,* E. mitis*,* E. mivati*,* E. brunetti*,* and E. tenella* [28, 31]. However, today’s diclazuril doses reached 5 to 10 ppm for prophylactic and therapeutic efficacy when used as water-soluble formulation (Diclosol 1%) and a feed additive formulation (Clinacox, 0.5%) that both 5 ppm and 10 ppm dosages showed the same efficacy against an experimental infection by *Eimeria* [30]. In the field trial diclazuril administered 2.5 ppm for 2 consecutive days with the instant bloody drops appeared, while the semi field administered diclazuril 1.25 ppm continuously for seven days prior to infection and 2.5 ppm two days after clinical signs showed high efficacy of DZ against coccidiosis [32]. When using diclazuril to treat *E. tenella* infection in chickens through drinking water at different concentrations (0.25, 0.5, or 1 mg / liter), the improvement increased with increasing the drug concentration, and the resistance index to the infection increased as well with increasing the rate diclazuril dose [33]. Generally, diclazuril has effective anticoccidial effect in the treatement and control so when used diclazuril as preventive treatment to coccidian [34]. Nano-emulsion encapsulation of bioactive substances improves their solubility, controlled release and absorption in the gastrointestinal system, and cell absorption [35, 36].

In the current study, two forms of diclazuril; normal and nanoemulsion forms were applied in two programs to control coccidiosis in chicken; therapeutic and prophylactic trials. Over all, the DZ nanoparticles had advantages more than normal form of DZ. The applied dose of DZN (2.5ppm) was a quarter of the normal form of DZ (10ppm). The nanoparticles materials reported enhancing the performance of treated chickens and significant antimicrobial activities [37–39]. Moreover, the nanotech could improve drugs stability, delivery, and the cellular uptake of nutrients and bioactive compounds, by protecting them from the stomach environment, releasing them in an intestinal environment, and consequently increasing their absorption [40]. Herein, DZN at 1/4 of normal DZ achieved the same results of DZ at 10ppm.

In this study, the BW and BWG declined severely in the infected untreated group while having no effect on the uninfected untreated group. However, at the fifth day after infection, the therapeutic groups considerably decreased but these groups had recovered, regained, and made up for the loss in BW but its BW are still smaller than the uninfected untreated group and much more than the infected untreated group. These results were similar to the findings of [41]. Due to decreased feed consumption, intestinal damage, and less efficient nutrient digestion, BW and BWG have decreased [42]. The Eimeria parasite infects the chicken’s intestine and impairs intestinal function All of these factors contributed to a significant drop in bloody diarrhea, a reduction in lesion scoring, a decrease in the number of oocysts shed daily and different histological findings between the treated and control groups. The coccidian replication in the treated groups was moderate to limited, and there were degenerative alterations like hyperplasia of the mucosal epithelium and leukocytic inflammatory infiltration with macrophages [43]. Diclazuril caused lymphocytes to destroy all intracellular developmental stages of *E. tenella*, whether they were sexual or asexual stages. the efficiency of DZ in drinking water for only 2 successive days at the 5th day post infection as a method of controlling avian coccidiosis was confirmed by the reduced number of oocysts recovered from feces, the lower macroscopic lesion score and the improved performance of chickens [18, 30, 44]. Diclzuril treatments caused an improvement in growth, due to reduced intestinal lesion scores demonstrating to upgrade gut health [45, 46].

The CBC in the infected groups (therapeutic groups G3, G4) was sharply decreased, as a result of second generation schizonts rupturing, resulting in substantial damage to the mucosal blood vessels and blood loss [47]. These findings are similar to Ellakany et al. 2011 and El-Maddawy et al. 2022 [48, 49]. Meanwhile, these indices were significantly increased in in the prophylactic groups (G5 & G6).

Moreover, liver and kidney functions in the infected therapeutic groups showed levels somewhat high if compared to negative control group. These results were in accordance with Shekhar et al. 2018 and El-Shazly et al. 2020 [50, 51]. These high levels could be related to coccidiosis-related hemorrhage as compensation for blood loss [52]. It’s important to ensure that the diclazuril still safe in nano emulsion form as safe in ordinary form so, liver and kidney functions tests showed no any adverse changes. These results of kidney and liver function ensure the safety of diclazuril on bird health. Also, the European Commission, the Panel on Additives and Products or Substances used in Animal Feed (FEEDAP) approved diclazuril from Coxiril^®^ as the potential to control coccidiosis in layer chickens at a minimum concentration of 0.8 mg/kg complete feed.

Over all, the DZ nanoparticles have advantageous to normal form of DZ. The applied dose of DZN (2.5ppm) is a quarter of the normal form of DZ (10ppm). The nanoparticles materials reported enhancing the performance of treated chickens and significant antimicrobial activities [37–39]. Moreover, the nanotech could improve drugs stability, delivery, and the cellular uptake of nutrients and bioactive compounds, by protecting them from the stomach environment, releasing them in an intestinal environment, and consequently increasing their absorption [40]. Confirming the nanotechnology efficacy achieved body weight and chicken productivity. Where, the supplementation of temulawak nanoemulsion to feed of broilers at rate of 4 mg/kg BW improved broiler chickens’ productivity [53]. Also, supplementation of selenium nanoparticles-loaded to chitosan improves chicken meat quality [54].

## Conclusions

Here, DZN at 1/4 of standard DZ yielded the same efficacy of DZ at 10ppm. As a result, the current study demonstrates that employing diclazuril nanoemulsion is sufficient to manage coccidiosis in chcikens at low dosages as compared to the standard form of diclazuril, with no adverse effects on diclazuril safety. So, this DZN needs to evaluate in the field to establish efficacy and safety.

## Data Availability

Data is provided within the manuscript or supplementary information files.

## References

[1] Noack S, Chapman HD, Selzer PM. Anticoccidial drugs of the livestock industry. Parasitol Res. 2019;118:2009–26. 10.1007/s00436-019-06343-5. 31152233 10.1007/s00436-019-06343-5 PMC6611755

[2] Imran A, Alsayeqh A. Anticoccidial Efficacy of Citrus sinensis essential oil in Broiler Chicken. Pakistan Veterinary J. 2022;42:461–6.

[3] Nahed A, Abd El-Hack ME, Albaqami NM, Khafaga AF, Taha AE, Swelum AA, El-Saadony MT, Salem HM, El-Tahan AM, AbuQamar. S F. Phytochemical control of poultry coccidiosis. A review. Poult Sci. 2022;101:101542. 34871985 10.1016/j.psj.2021.101542 PMC8649401

[4] Mohammed BR, Sunday OS. An overview of the prevalence of avian coccidiosis in poultry production and its economic importance in Nigeria. Vet Res Int. 2015;3(3):35–45.

[5] Teng PY, Yadav S, de Castro FLS, Tompkins YH, Fuller AL, Kim WK. Graded Eimeria challenge linearly regulated growth performance, dynamic change of gastrointestinal permeability, apparent ileal digestibility, intestinal morphology, and tight junctions of broiler chickens. Poult Sci. 2020;99:4203–16. 32867964 10.1016/j.psj.2020.04.031 PMC7598010

[6] Dalloul RA, Lillehoj HS. Poultry coccidiosis: recent advancements in control measures and vaccine development. Expert Rev Vaccines. 2006;5(1):143–63. 10.1586/14760584.5.1.143. 16451116 10.1586/14760584.5.1.143

[7] Fornace KM, Clark EL, Macdonald SE, Namangala B, Karimuribo E, Awuni JA, Thieme O, Blake DP, Rushton J. Occurrence of Eimeria species parasites on small-scale commercial chicken farms in Africa and indication of economic profitability. PLoS ONE. 2013;8(12):e84254. 24391923 10.1371/journal.pone.0084254 PMC3877271

[8] Pop L, Györke A, Tǎbǎran AF, Dumitrache MO, Kalmár Z, Magdaş C, Mircean V, Zagon D, Balea A, Cozma V. Effects of artemisinin in broiler chickens challenged with Eimeria acervulina, E. maxima and E. tenella in battery trials. 2015 Vet Parasitol 214(3–4): 264–71. 10.1016/j.vetpar.2015.10.011 10.1016/j.vetpar.2015.10.011 26518641

[9] Conway D, Mathis G, Johnson J, Schwartz M, Baldwin C. Efficacy of diclazuril in comparison with chemical and ionophorous anticoccidials against Eimeria spp. in broiler chickens in floor pens. Poult sci. 2001;80(4):426–30. 11297280 10.1093/ps/80.4.426

[10] Stock ML, Elazab ST, Hsu WH. Review of triazine antiprotozoal drugs used in veterinary medicine. J Vet Pharmacol Th. 2017;41(2):184–94. 10.1111/jvp.12450. 10.1111/jvp.12450 28833212

[11] Zhou BH, Liu LL, Liu J, Yuan FW, Tian EJ, Wang HW. Effect of Diclazuril on the Bursa of Fabricius morphology and SIgA expression in chickens infected with Eimeria tenella. 2015. Korean J Parasitol. 2015;53(6):675–82. 26797433 10.3347/kjp.2015.53.6.675 PMC4725230

[12] Vanparijs O, Marsboom R, Deplentar L. Diclazuril, a new broad spectrum anticoccidial drug in chickens. 1. Dose titration studies and pilot floor pen trials. Poult Sci. 1989;68:489–95. 2748496 10.3382/ps.0680489

[13] Abbas R, Iqbal Z, Sindhu Z-D, Khan M, Arshad M. Identification of cross resistance and multiple resistance in Eimeria tenella field isolates to commonly used anticoccidials in Pakistan. 2008. J Appl Poult Res. 2008;17(3):361–8.

[14] Elgadir MA, Uddin MS, Ferdosh S, Adam A, Chowdhury AJK, Sarker MZI. Impact of chitosan composites and chitosan nanoparticle composites on various drug delivery systems. J Food Drug Anal. 2015;23:619–29. 28911477 10.1016/j.jfda.2014.10.008 PMC9345468

[15] Youssef IMI, Abdel-Razik AH, Aboelhadid SM, Arafa WM, Shany SA, Abdel-Daim ASA. Comparative effects of dietary saponin and probiotic supplementation on performance, carcass traits and intestinal histomorphology of broilers challenged with E. tenella. 2021. Iran J Appl Anim Sci 11:147–59.

[16] Zhang S, Zhang M, Fang Z, Liu Y. Preparation and characterization of blended cloves/cinnamon essential oil nanoemulsions. LWT Food Sci Technol. 2017;75:316–22.

[17] Ewais O, Abdel-Tawab H, El-Fayoumi H, Aboelhadid SM, Al-Quraishy S, Falkowski P, Abdel-Baki AS. Administration of Ethanolic Extract of Spinacia oleracea Rich in Omega-3 improves oxidative stress and goblet cells in broiler chickens infected with Eimeria tenella. Molecules. 2023;28(18):6621. 10.3390/molecules28186621. PMID: 37764396; PMCID: PMC10534835. 37764396 10.3390/molecules28186621 PMC10534835

[18] El-Sawah AA, Aboelhadid SM, El-Nahass EN, Helal HE, Korany AM, El-Ashram S. Efficacy of probiotic Enterococcus faecium in combination with diclazuril against coccidiosis in experimentally infected broilers. 2020 J Appl Microbiol Oct;129(4):1020–8. 10.1111/jam.14691. Epub 2020 May 26. PMID: 32364304. 10.1111/jam.14691 32364304

[19] Morehouse NF, Baron RR. Coccidiosis: evaluation of coccidiostats by mortality, weight gains, and fecal scores. Exp Parasitol. 1970;28(1):25–9. 5459869 10.1016/0014-4894(70)90062-7

[20] Johnson J, Reid WM. Anticoccidial drugs: lesion scoring techniques in battery and floor-pen experiments with chickens. Exp Parasitol, 1970, 28(1): 30–6. 10.1016/0014-4894(70)90063-9 5459870

[21] Lillehoj HS, Ruff MD. Comparison of disease susceptibility and subclass-specific antibody response in SC and FP chickens experimentally inoculated with Eimeria tenella, E. acervulina, or E. maxima. 1987. Avian Dis. 31:112–119. 3579780

[22] Ma D, Ma C, Pan LL, Yan J, Hong J, Cai H, Ren X. Vaccination of chickens with DNA vaccine encoding Eimeria acervulina 3-1E and chicken IL-15 offers protection against homologous challenge. Exp Parasitol. 2011;127(1):208–14. 20688059 10.1016/j.exppara.2010.07.015

[23] Voeten AC, Braunius WW, Orthel FW, van Rijen MA. Influence of coccidiosis on growth rate and feed conversion in broilers after experimental infections with *E*imeria acervulina and *E*imeria maxima. Vet Q. 1988;10:256–64. 10.1080/01652176.1988.9694182 3218068

[24] Bancroft J, Gamble A. Theory and Practice of Histological Techniques, 6th ed. 2008.ChurchillLivingstone, Edinburgh, UK London, UK Melbourne, Australia; New York, NY, USA.

[25] Reitman S, Frankel S. A colorimetric method for determination of serum glutamic oxaloacetic transaminase and serum glutamic pyruvic transaminase. Am J clin Path, 1957, 25–56. 10.1093/ajcp/28.1.56 13458125

[26] Gilbert ER, Cox CM, Williams PM, McElroy AP, Dalloul RA, Ray WK, Barri A, Emmerson DA, Wong EA, Webb KE Jr. Eimeria species and genetic background influence the serum protein profile of broilers with coccidiosis. PLoS ONE. 2011;6(1):e14636. 10.1371/journal.pone.0014636. 10.1371/journal.pone.0014636 PMC3031500 21297942

[27] Chapman HD. Eimeria tenella, E., acervulina and E. maxima: studies on the development of resistance to diclazuril and other anticoccidial drugs in chickens. Parasitol. 1989;99(2):189–92. 10.1017/s0031182000058625 2556680

[28] Vanparijs O, Desplenter L, Marsboom R. Diclazuril, a new broad spectrum anticoccidial drug in chickens: 1. Dose titration studies and pilot floor pen trials. Poult Sci, 1989b, 68 (4):489–95. 10.3382/ps.0680489 2748496

[29] Awaad MH, Afify MA, Zouelfakar SA, Hilali MA. Anticoccidial efficacy of steroidal sapogenins (organic coccidiostate) in broiler chickens (semi-field and field trials). Egypt Vet Med Soci Parasitol J. 2003;1(1):123–36.

[30] El-Banna HA, El-Bahy MM, ElZorba HY, El-Hady M. Anticoccidial efficacy of drinking water soluble diclazuril on experimental and field coccidiosis in broiler chickens. J Vet Med Physiol Pathol Clin Med. 2005;52(6):287–91. 10.1111/j.1439-0442.2005.00727. 10.1111/j.1439-0442.2005.00727.x 16050910

[31] Vanparijs O, Desplenter L, Marsboom R. Efficacy of diclazuril in the control of intestinal coccidiosis in rabbits. 1989a. Vet Parasitol, 1989a, 34(3):185–90. 10.1016/0304-4017(89)90049-6 2617823

[32] Amer MM, Wafaa AA, ElGhany, Aziza M, Amer, Hanafei AEA, Zohair GA. The efficacy of diclazuril (liquid formulation) in the prevention and control of coccidiosis in broiler chicken. Bs. Vet. Med. J. November 5th Scientific Conference. 96101. 2007. Beni-suef Veterinary Medicalِ Journal.

[33] Jiang-Zhong QI. Study of the efficacy of diclazuril solution in Eimeria tenella inoculated chicks. Zhejiang Nongye Kexue. 1999;1:44–6.

[34] Habibi H, Firouzi S, Nili H, Asadi MRSL, Daneshi S. Anti coccidial effects of herbal extracts on *Eimeria tenella* infection in broiler chickens: in-vitro and in-vivo study. J 2014 Paras Dis. 2014;40(2):401–7. 10.1007/s.12639-014-0517-4. 10.1007/s12639-014-0517-4 PMC4927499 27413312

[35] Chen L, Remondetto GE, Subirade M. Food protein-based materials as nutraceutical delivery systems. Trends Food Sci Technol. 2006;17:272–83. 10.1016/j.tifs.2005.12.011.

[36] McClements DJ, Rao J. Food-grade nanoemulsions: formulation, fabrication, properties, performance, biological fate, and potential toxicity. Crit Rev Food Sci Nutr. 2011;51:285–330. 10.1080/10408398.2011.559558. 21432697 10.1080/10408398.2011.559558

[37] Pineda L, Chwalibog A, Sawosz E, Lauridsen C, Engberg R, Elnif J, Hotowy A, Sawosz F, Gao Y, Ali A. Effect of silver nanoparticles on growth performance, metabolism and microbial profile of broiler chickens. Arch Anim Nutr. 2012;66:416–29. 22889095 10.1080/1745039X.2012.710081

[38] Sahoo A, Swain R, Mishra SK. Effect of inorganic, organic and nano zinc supplemented diets on bioavailability and immunity status of broilers. Int J Adv Res, 2014, *2*, 828–37.

[39] Scott A, Vadalasetty K, Sawosz E, Łukasiewicz M, Vadalasetty R, Jaworski S, Chwalibog A. Effect of copper nanoparticles and copper sulphate on metabolic rate and development of broiler embryos. Anim Feed Sci Technol. 2016;220:151–8.

[40] Hill EK, Li J. Current and future prospects for nanotechnology in animal production. J Anim Sci Biotechnol. 2017;8:1–13. 28316783 10.1186/s40104-017-0157-5 PMC5351054

[41] Elagib HAA, El-Amin WIA, Elamin KM, Malik HEE. Effect of dietary garlic (*Allium sativum*) supplementation as feed additive on broiler performance and blood profile. J Anim Sci Ad. 2013;3(2):58–64. 10.5455/jasa.20130219104029.

[42] Walk CL, Cowieson AJ, Remus JC, Novak CL, McElroy AP. Effects of dietary enzymes on performance and intestinal goblet cell number of broilers exposed to a live coccidia oocyst vaccine. Poult Sci. 2011;90(1):91–8. 10.3382/ps.2010-00760. 21177448 10.3382/ps.2010-00760

[43] Choi J, Ko H, Tompkins YH, Teng PY, Lourenco JM, Callaway TR, Kim WK. Effects of Eimeria tenella infection on key parameters for feed efficiency in broiler chickens. Anim (Basel). 2021;11(12):3428. 10.3390/ani11123428. 10.3390/ani11123428 PMC8697946 34944205

[44] Assis RCL. Cury^I^ MC, Luns^II^ FD, Assis RL. Anticoccidial efficacy of drinking water soluble diclazuril in the control of Eimeria acervulina oocysts on experimentally-infected broiler chickens. 2012. Arq. Bras. Med. Vet. Zootec. 64 (5) • Oct 2012 • 10.1590/S0102-09352012000500016

[45] El-Azm IMA, El-Hamid HAS, Ellakany HF, El-Shall NA. Sensitivity of two local field isolates of Eimeria tenella to maduramycin and diclazuril. Zagazig Vet J. 2010;38:8–18.

[46] Iraee AH, Iraee AM, Youssefi MR, Tabari AM. Growth performance parameters in chicken experimental coccidiosis treated with diclazuril and clopidol: the need for assessing new anticoccidial resources. Iran J Vet Med. 2015;9:189–94.

[47] Nayak D, Rai P. Hemogram of chickens experimentally infected with eimeria species. Indian J Vet Med. 1985;5:42–3.

[48] Ellakany HF, Abuakkada SS, Oda SS, El-Sayed YS. Influence of low levels of dietary aflatoxins on eimeria tenella infections in broilers. Trop Anim Health Prod. 2011;43:249–57. 20737287 10.1007/s11250-010-9685-0

[49] El-Maddawy ZK, El-Sawy AEF, Ashoura NR, Aboelenin SM, Soliman MM, Ellakany HF, Elbestawy AR, El-Shall NA. Use of Zinc Oxide Nanoparticles as Anticoccidial Agents in Broiler Chickens along with Its Impact on Growth Performance, Antioxidant Status and Hematobiochemical Profile. 2022. Life (Basel). Jan 5;12(1):74. 10.3390/life12010074. PMID: 35054467; PMCID: PMC8779200. 10.3390/life12010074 PMC8779200 35054467

[50] Shekhar S, Shula S, Bhatt P, Kumar M, Bisth D. Comparative efficacy of melia azedarach extracts with amprolium against experimentally induced coccidiosis in broiler. Int J Curr Microb Appl Sci, 2018, *7*, 2656–63.

[51] El-Shazly KA, El-Latif AA, Abdo W, El-Morsey A, El-Aziz MIA, El-Mogazy H. The anticoccidial activity of the fluoroquinolone lomefloxacin against experimental Eimeria tenella infection in broiler chickens. Parasitol Res. 2020;119:1955–68. 32399722 10.1007/s00436-020-06692-6

[52] Adamu M, Boonkaewwan C, Gongruttananun N, Vongpakorn M. Hematological, biochemical and histopathological changes caused by coccidiosis in chickens. Agric Nat Resour. 2013;47:238–46.

[53] Orinetha J, Salsabil JK, Putri SM, Pratama AM. Temulawak (Curcuma Xanthorrhiza Roxb.) Nanoemulsion can be substituted as natural growth promoter in broiler chickens. Pak Vet J. 2022;42(3):409–13. 10.29261/pakvetj/2022.022.

[54] Khan I, Zaneb H, Masood S, Ashraf S, Rehman HF, Rehman HU, Ahmad S, Taj R, Salahuddin, Rahman SU. Supplemental selenium nanoparticles-loaded to Chitosan improves meat quality, pectoral muscle histology, tibia bone morphometry and tissue mineral retention in broilers. 2022. Pak Vet J, 42(2): 236–40. 10.29261/pakvetj/2022.007

